# Pericyte, but not astrocyte, hypoxia inducible factor-1 (HIF-1) drives hypoxia-induced vascular permeability in vivo

**DOI:** 10.1186/s12987-021-00302-y

**Published:** 2022-01-15

**Authors:** Julia Baumann, Chih-Chieh Tsao, Shalmali Patkar, Sheng-Fu Huang, Simona Francia, Synnøve Norvoll Magnussen, Max Gassmann, Johannes Vogel, Christina Köster-Hegmann, Omolara O. Ogunshola

**Affiliations:** 1 Institute of Veterinary Physiology and Center for Clinical Studies, University of Zurich, Vetsuisse Faculty, Winterthurerstrasse 260, CH-8057 Zurich, Switzerland; 2 Institute of Medical Biology, Faculty of Health Sciences, UiT-The Arctic University of Norway, Tromsø, Norway

**Keywords:** Blood–brain barrier, Neurovascular unit, Perivascular signaling, Vascular remodeling, Cerebral edema, Tight junctions, Endothelium

## Abstract

**Background:**

Ways to prevent disease-induced vascular modifications that accelerate brain damage remain largely elusive. Improved understanding of perivascular cell signalling could provide unparalleled insight as these cells impact vascular stability and functionality of the neurovascular unit as a whole. Identifying key drivers of astrocyte and pericyte responses that modify cell–cell interactions and crosstalk during injury is key. At the cellular level, injury-induced outcomes are closely entwined with activation of the hypoxia-inducible factor-1 (HIF-1) pathway. Studies clearly suggest that endothelial HIF-1 signalling increases blood–brain barrier permeability but the influence of perivascular HIF-1 induction on outcome is unknown. Using novel mouse lines with astrocyte and pericyte targeted HIF-1 loss of function, we herein show that vascular stability in vivo is differentially impacted by perivascular hypoxia-induced HIF-1 stabilization.

**Methods:**

To facilitate HIF-1 deletion in adult mice without developmental complications, novel Cre-inducible astrocyte-targeted (GFAP-CreER^T2^; HIF-1α^fl/fl^ and GLAST-CreER^T2^; HIF-1α^fl/fl^) and pericyte-targeted (SMMHC-CreER^T2^; HIF-1α^fl/fl^) transgenic animals were generated. Mice in their home cages were exposed to either normoxia (21% O_2_) or hypoxia (8% O_2_) for 96 h in an oxygen-controlled humidified glove box. All lines were similarly responsive to hypoxic challenge and post-Cre activation showed significantly reduced HIF-1 target gene levels in the individual cells as predicted.

**Results:**

Unexpectedly, hypoxia-induced vascular remodelling was unaffected by HIF-1 loss of function in the two astrocyte lines but effectively blocked in the pericyte line. In correlation, hypoxia-induced barrier permeability and water accumulation were abrogated only in pericyte targeted HIF-1 loss of function mice. In contrast to expectation, brain and serum levels of hypoxia-induced VEGF, TGF-β and MMPs (genes known to mediate vascular remodelling) were unaffected by HIF-1 deletion in all lines. However, in agreement with the permeability data, immunofluorescence and electron microscopy showed clear prevention of hypoxia-induced tight junction disruption in the pericyte loss of function line.

**Conclusion:**

This study shows that pericyte but not astrocyte HIF-1 stabilization modulates endothelial tight junction functionality and thereby plays a pivotal role in hypoxia-induced vascular dysfunction. Whether the cells respond similarly or differentially to other injury stimuli will be of significant relevance.

**Supplementary Information:**

The online version contains supplementary material available at 10.1186/s12987-021-00302-y.

## Background

Separation of the brain parenchyma from the majority of substances circulating in the blood stream is critical for neuronal homeostasis and brain functionality. The blood–brain barrier (BBB) performs this role. The BBB is a complex dynamic structure consisting of microvascular endothelial cells that line the vessel wall, astrocyte endfeet, pericytes, as well as the basal lamina [1]. These components interact in concert to maintain the characteristic low paracellular flux of the brain vascular system, presence of specific transporters that facilitate entry of essential nutrients and metabolizing enzymes that remove toxic and/or waste products [2, 3]. The importance of the perivascular cells, astrocytes and pericytes, cannot be overestimated. Astrocytes have been long known to support surrounding neurons and the vasculature. They buffer pH and ion concentrations, provide nutrients, modulate neurotransmitter release and uptake, and participate in brain cell communication during both development and adulthood [1, 4]. They also play pivotal roles in inducing the barrier phenotype of cerebrovascular endothelial cells during development and maintaining barrier stability through release of soluble factors [4, 5]. Pericytes intimately contact the vascular endothelium by sharing a basement membrane and also impact many aspects of vascular functionality [6]. Elegant studies using pericyte-deficient mouse models conclusively showed pericytes regulate functional aspects of the embryonic and adult BBB in vivo [7–9]*.* Indeed, they strongly influence expression of BBB-specific genes and proteins, regulate vascular tone as well as polarize astrocyte end-feet surrounding CNS blood vessels, underlying their important role in both endothelial and astrocyte functional integration [6, 10]. Perivascular cells are a rich source of growth factors and potential permeability-modulating proteins, and their ability to signal to the endothelium is thought to occur predominantly via soluble and solid phase factors as well as cognate receptors [11–14].

Many cerebrovascular and neurodegenerative diseases are characterized by impaired O_2_ delivery (hypoxia) to the cerebral tissue and functional impairment of the neurovascular unit (NVU) [15]. Due to its central role in preserving the brain microenvironment and supporting NVU function, it is unsurprising that a disturbed barrier only exaggerates injury progression. Despite this clarity however, our understanding of BBB regulation during diseases remains rudimentary resulting in a shortfall of progress in the fight against vascular dysfunction. We are convinced that better understanding of perivascular cell signalling will provide unparalleled insight as these cells directly impact vascular stability and NVU functionality as a whole. Clearly perivascular cell adaptation to injury alters not only their signalling profiles and secretion of diverse injury-regulated factors [6, 16], but also thereby modifies cell–cell interactions and crosstalk [1, 17]. Identifying key drivers of such responses, and how they influence outcome, could thus provide new ways to modulate barrier function.

Hypoxia inducible factor-1 (HIF-1) is a master regulator of injury-induced cellular responses, and activation of this pathway is critical for rapid adaptation to adverse environmental conditions [18, 19]. However HIF-1 is a double-edged sword, being protective in some instances but detrimental in others when it induces apoptotic pathways or activating other mechanisms that lead to cell death [20]. Generally, whether HIF-1 stabilization has positive or negative effects depends largely on injury severity and the cell type affected. At the level of the brain endothelium, most data supports the notion that endothelial HIF-1 stabilization induces BBB permeability. In vitro data showed downstream HIF-1 target genes such as vascular endothelial growth factor (VEGF) and matrix metalloproteinases (MMPs) disturbs expression, localization and phosphorylation of tight junction (TJ) proteins thereby preventing tight endothelial contacts [20–23]. In vivo studies also lend weight to this argument. In rat models of cerebral focal ischemia pharmacological inhibition of HIF-1 reduced edema formation and infarct volume, and protected the BBB [24–27]. Using endothelium-specific HIF-1-deficient mice, Zhang et al*.* directly demonstrated the disruptive nature of endothelial HIF-1 induction during diabetic stroke [28]. HIF-1 is also implicated in mediating BBB disruption in other brain injuries including traumatic brain injury [29] and subarachnoid haemorrhage [30]. Although the endothelium itself is a major source of HIF-1-induced negative effects, consequences of HIF-1 stabilization by perivascular cells are also of high relevance as these cells are significant stores and sites of release of HIF-1 induced permeabilizing factors [11, 13, 14].

We asked if astrocyte or pericyte HIF-1 deletion alters endothelial functionality and has similar or differential effects on BBB stability using novel mouse lines generated for the purpose. Surprisingly, using two independent mouse lines we noted that blocking astrocyte HIF-1 did not alter vascular stability. In contrast pericyte targeted HIF-1 loss of function abrogated both hypoxia-induced vascular alterations and barrier dysfunction.

## Methods

### Generation of transgenic astrocyte- and pericyte-targeted HIF-1 Loss of function (LoF) mice

**Fig. 1:**
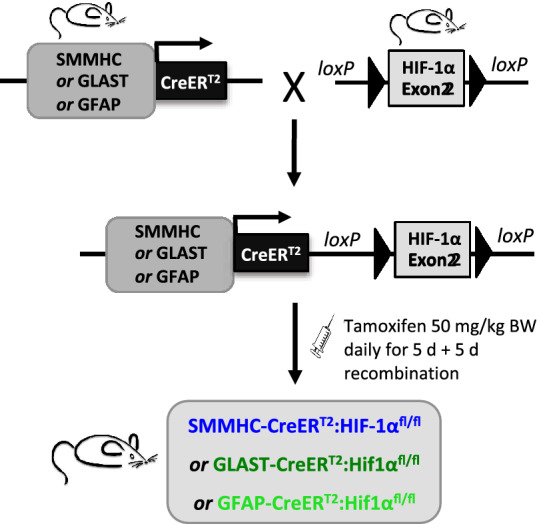
Generation of transgenic perivascular mouse lines. Schematic overview of the generation of transgenic mouse lines with conditional (CreER^T2^) astrocyte- (GFAP, GLAST) or pericyte-targeted (SMMHC) HIF-1 LoF. Excision of floxed HIF-1α exon 2 was induced by intraperitoneal injection of tamoxifen (50 mg/kg body weight) over 5 consecutive days followed by a 5 day recombination period

Mice were standardly housed at a constant temperature of approx. 22 °C under a 12 h dark/light cycle with unlimited access to water and normal chow (65% carbohydrate, 22% protein, and 12.5% fat). The cages were equipped with nesting material and housing structures**.** All animal procedures were performed in accordance with Swiss animal protection laws and approved by the University of Zurich Institutional Animal Care guidelines. For clarity, pericyte data are always presented in blue graphs while astrocyte data is green. Cell-specific HIF-1 loss of function (LoF) mice were generated using homozygous floxed HIF-1α^fl/fl^ mice in which exon 2 is floxed [31] (kindly provided by Dr. Randall Johnson, University of Cambridge, UK). Notably, the HIF-1α exon 2 is critical for the dimerization of HIF-1α with HIF-1β and subsequent HIF-1 transcriptional activity. Thus excision of exon 2 via Cre recombinase prevents both dimerization and transcriptional activation resulting in HIF-1 LoF. Astrocyte-specific HIF-1 LoF animals were generated by crossing these mice with a line expressing a Cre recombinase fusion protein having a modified estrogen receptor binding domain (CreER^T2^) under the control of either the glial fibrillary acidic protein (GFAP) [32] or glutamate aspartate transporter (GLAST) [33] promoter to obtain Cre heterozygous floxed mice, namely GFAP-CreER^T2^; HIF-1α^fl/fl^ or GLAST-CreER^T2^; HIF-1α^fl/fl^ respectively (Fig. 1). Both male and female animals were included in the analysis. Pericyte-targeted HIF-1α LoF was obtained by crossing the same floxed animal with a smooth muscle myosin heavy chain promoter (SMMHC)-CreER^T2^ line kindly provided by Dr. Stephan Offermanns, Max Planck Institute, Germany (Fig. 1). Cre-mediated recombination in these mice has been shown to occur mainly in microvascular pericytes and to a lesser degree in smooth muscle cells of larger vessels [34]. Other studies confirm the SMMHC-CreER^T2^ system localizes mainly to pericytes in the capillary bed [35, 36] a fact confirmed by our observation that HIF-1α LoF did not impact hypoxic water content or Evans Blue leakage in organs outside the CNS (data not shown). Only male littermates were used as SMMHC-CreER^T2^ is located on the Y chromosome [34]. It is also important to note that all lines had different genetic backgrounds despite being crossed to the same Cre line. Standard mouse genotyping was performed on DNA isolated from ear biopsies using single or duplex polymerase chain reaction (PCR) with primers against HIF-1α and the specific Cre recombinase (commercially obtained from Microsynth AG, Switzerland). Primer sequences, concentrations and annealing temperatures are indicated in Table 1. The PCRs were standardly run with 35 cycles and an extension temperature of 72 °C.

**Table 1:** Overview of primers and conditions. *C.s.: Cre-ER^T2^ specific primers

Genotype	Primer sequence	Primer conc	Annealing temp
HIF-1α	Fw. 5’-GCAGTTAAGAGCACTAGTTG-3’ Rv. 5’-GGAGCTATCTCTCTAGACC-3’	400 nM 400 nM	57 °C
SMMHC	Fw. 5’-TGACCCCATCTCTTCACTCC-3’ Rv. 5’-AACTCCACGACCACCTCATC-3’ *C.s. 5’-AGTCCCTCACATCCTCAGGTT-3’	600 nM 300 nM 600 nM	58 °C
GFAP	Fw. 5’-CCTGGAAAATGCTTCTGTCCG-3’ Rv. 5’-CAGGGTGTTATAAGCAATCCC-3’	200 nM 200 nM	59 °C
GLAST	Fw. 5’-GAGGCACTTGGCTAGGCTCTGAGGA-3’ Rw. 5’-GAGGAGATCCTGACCGATCAGTTGG-3’ *C.s. 5’-GGTGTACGGTCAGTAAATTGGACAT-3’	400 nM 400 nM 400 nM	58 °C

### Tamoxifen-induced HIF-1 allele recombination and confirmation of deletion

Animals were randomly assigned to groups by the study director. Subsequent experiments and analyses were performed double-blinded. Tamoxifen was used to induce excision of HIF-1α exon 2 in 8–12 week old adult mice. Briefly, tamoxifen (Sigma-Aldrich, Switzerland) was dissolved in a sunflower oil/ethanol (10:1) mixture at 100 mg/ml. Animals were injected once a day intraperitoneally (50 mg/kg body weight) for 5 consecutive days and experiments started only after 5 subsequent days had elapsed to allow complete recombination (Additional file 1: Fig. S1A). The tamoxifen stock solution was adjusted with oil to a final volume of 40 µl for all injections, control mice were injected with 40 µl sunflower oil/ethanol mix only. Exon deletion was confirmed using genomic DNA isolated from cortical tissue or isolated microvessels. Standard PCR for floxed HIF-1α (35 cycles, 65 °C elongation) was performed on 50 ng of genomic DNA using the following primers (200 nm final concentration): HIF-1α^flox/flox^ forward 5’-GGGATGAAAACATCTGCTTTGGA-3’ and reverse 5’-TGTGTTGGGGCAGTACTGG-3’ and β-actin forward 5’-CTGGCTCCTAGCACCATGAAG-3’ and reverse 5’-GCCACCGATCCACACAGAGT-3’.

### Western Blot for cytosolic and nuclear Cre recombinase localisation

Half brains were homogenized with a dounce homogenizer in ice cold buffer (0.27 M sucrose, 2 mM EDTA pH8, 600 mM KCl, 15 mM NaCl, 15 mM HEPES) supplemented with 1 mM phenylmethansulfonyl fluoride (PMSF, Sigma-Aldrich, Switzerland), 1 mM sodium orthovanadate (NaV, Sigma-Aldrich, Switzerland), and proteinase inhibitor cocktail (Calbiochem, Germany). The cell suspension was layered onto a sucrose cushion (30% w/v sucrose, 2 mM EDTA pH8, 600 mM KCl, 15 mM NaCl, 15 mM HEPES) and centrifuged at 1500 g for 10 min at 4 °C. The cytosolic fraction was collected and the nuclear pellet resuspended in extraction buffer (20 mM HEPES, 400 mM NaCl, 1 mM EDTA pH8) supplemented with 1 mM PMSF, 1 mM NaV and proteinase inhibitor cocktail. After 15 min incubation on ice the nuclear fraction was obtained by centrifugation (15′000 g for 5 min) at 4 °C. Proteins (30 µg) were separated by SDS-PAGE then transferred to nitrocellulose membranes (Amersham™ 0.45 µm, Sigma-Aldrich, Switzerland). After blocking in 5% non-fat dry milk in TBS, membranes were incubated with primary antibodies against Cre (1:1000, MAB3120, Sigma-Aldrich, Switzerland) and β-actin (1:5000, A5441, Sigma-Aldrich, Switzerland). Membranes were then incubated with horseradish peroxidase-conjugated secondary antibodies for 1 h at RT. Bands were detected using a luminescent image analyzer (Fujifilm, LAS-3000, Switzerland).

### Hypoxic exposure

Mice in their home cages were exposed to either normoxia (21% O_2_) or hypoxia (8% O_2_) for 96 h in an oxygen-controlled humidified glove box (Coy Laboratories, USA) with unlimited access to food and water (standard chow). Temperature (20–24 °C) and relative humidity (45–60%) were also computer controlled. Post euthanisation, blood samples were sampled by heart puncture and hematocrit was used to confirm the hypoxic impact. As expected, all animals demonstrated similar hemoconcentration that was unaffected by tamoxifen treatment (Additional file 1: Fig. S1B).

### Primary cell isolation

All cell culture reagents were obtained from Gibco (Thermo Fischer Scientific, Switzerland) unless otherwise indicated. Astrocyte isolation was performed according to an in-house protocol. In short, isolated cortices were mechanically dissociated then enzymatically digested in HBSS supplemented with 0.25% Trypsin, 10 mM EDTA and 1 mg DNaseI (Roche, Switzerland) at 37 °C for 30 min. The homogenate was separated by gradient centrifugation (22% BSA in PBS, 3000 g) and cells resuspended in DMEM supplemented with 10% horse serum, 1% Penicillin/streptomycin, 1% L-glutamine and 50 µg/ml gentamycin (AppliChem GmbH, Germany). Cells were seeded on collagen IV coated 24 well plates and media changed first after 48 h, then every 5 days until near confluency. Astrocytes were not passaged before use.

Pericytes were isolated as previous [37] with slight modifications. Cortices were homogenized and the tissue digested in DMEM buffer supplemented with 2 mg/ml collagenase-dispase, 10 μg/ml DNase I and 30U/ml papain for 60 min at 37 °C. The digested tissue was then homogenized by passing subsequently through 18 and 21 gauge needles. The homogenate was separated by gradient centrifugation (22% BSA in PBS) at 4000 g for 10 min. The obtained cell pellet was washed and after final centrifugation (700 g for 5 min), pericytes were plated on collagen coated petri dishes in selective media (DMEM containing 20% FBS, 50 μg/ml gentamycin sulfate and 2.5 μg amphotericin B) until confluency. After first passage cells were maintained on uncoated dishes without amphotericin and used at passage 1. Purity of all cultures was ≥ 98% as assessed by immunostaining for standard cell markers GFAP, GLAST, NG-2 and PDGFR-β as previous [37]. Iba1 and CD31 immunostaining further confirmed absence of contaminating microglia or endothelial cells.

### Quantitative Real-time PCR (qPCR)

Astrocytes and pericytes isolated from GLAST-CreER^T2^; HIF-1α^fl/fl^ and SMMHC-CreER^T2^; HIF-1α^fl/fl^ mice respectively, were treated with oil or tamoxifen (2 µM) during 48 h hypoxic exposure (1% O_2_) then harvested in TRIzol® reagent (Thermo Fisher Scientific, Switzerland). As shown previously [37], this time point ensures hypoxic target gene induction in these primary cells without the occurrence of cell death. Total RNA was isolated by PureLink® RNA Mini Kit (Invitrogen, USA) according to the manufacturer’s description. 1 μg of RNA was reverse transcribed (ImProm-II ReverseTranscriptase kit, Promega, Switzerland) and 20 ng cDNA used for Power Sybr® Green qPCR with an ABI 7500 Fast Real-Time PCR System (Applied Biosystems, Switzerland). QPCR primers were used at 100 nM final concentration: Glut1 forward 5’-GGGCATGATTGGTTCCTTCTC-3’ and reverse 5’-CAGGTTCATCATCAGCATGGA-3’; VEGF forward 5’-CGCAAGAAATCCCGGTTTAA-3’ and reverse 5’-CAAATGCTTTCTCCGCTCTAA-3’; β-actin forward 5’-CTGGCTCTAGCACCATGAAG-3’ and reverse 5’-GCCACCGATCCACACAGAGT-3’. All data were normalized to β-actin and fold changes were calculated based on the comparative ΔΔCt method.

### Water content assessment

At experimental end animals were euthanized by CO_2_ inhalation. Brain and other organs were quickly removed. Wet weights were measured then tissues dried at 85 °C for 72 h to obtain dry weight values. Water content was calculated as [(wet – dry weight)/wet weight] × 100% and graphically represented. Due to hypoxia-induced body weight loss, water content was normalized to end body weight in GLAST-CreER^T2^; HIF-1α^fl/fl^ mice [38].

### Tracer permeability assay

Tracer extravasation was used to assess vascular permeability as previously described [39]. Briefly, mice were anesthetized with isoflurane then injected with a mixture of 450 Da Lucifer yellow (6.25 mM, Sigma-Aldrich, Switzerland) and 70 KDa Texas red dextran (0.01875 mM, Invitrogen, USA) in 0.9% NaCl via the femoral vein. After circulation for 3 min mice were overdosed with ketamine/xylazine and transcardially perfused with ice-cold PBS for 10 min. Brains were harvested, weighed then homogenized in ice cold PBS. After centrifugation (10,000 g for 5 min) fluorescence intensity of the supernatants was measured (excitation 425 nm and emission 525 nm for Lucifer yellow; emission 595 nm and excitation 625 nm for Texas red dextran). Sham animals, injected with 0.9% NaCl only, were included in each experiment to control for background auto fluorescence. Brain fractions were calculated using the formula: (brain raw fluorescent units/ serum raw fluorescent units)/ brain wet weight [mg]. Due to hypoxia-induced body weight loss in GLAST-CreER^T2^; HIF-1α^fl/fl^ mice this formula was corrected for total blood volume of each animal at experimental end: (brain raw fluorescent units/ serum raw fluorescent units) / (brain wet weight [mg] x end blood volume [ml]).

### Evans Blue leakage assay

Animals were anesthetized then injected with 1% Evans blue dye (2 μg/g body weight) via the femoral vein. After 45 min circulation time, blood was collected via heart puncture and animals transcardially perfused with ice cold PBS for 10 min. Brains were then collected, rinsed in ice-cold PBS, blotted dry and weighed. Dye retained in the tissue was extracted with formamide (5 μl/mg tissue) for 72 h. Absorbance was measured at 620 nm and expressed as fold change of normoxic Ctrl mice.

### Immunofluorescence staining

Brain sagittal cryosections (20 µm) at 1.35–1.525 mm lateral to the midline were fixed in 4% paraformaldehyde for 10 min then incubated in 5% NGS in PBS for 60 min. Endogenous mouse IgG was blocked for 2 h with unconjugated goat anti-mouse IgG Fab Fragments (1:100, 20 µg/ml, 115,007, Jackson ImmunoResearch, UK). Sections were incubated overnight at 4 °C with antibodies against CD31 (1:1000, 5 µg/ml, 553,370, Millipore, USA), ZO-1 (1:100, 2.5 µg/ml, 61–7300, Invitrogen, USA), claudin-5 (1: 250, Invitrogen, USA), NG-2 (1:200, 5 µg/ml, AB5320, Millipore, USA), PDGFR-β (1:500, 0.4 µg/ml, sc-432, Santa Cruz, USA) or Cre recombinase (1:500, MAB3120, Millipore, USA) followed by secondary antibody for 1 h and counterstained with DAPI. Images were acquired with an epifluorescence microscope (Carl Zeiss, Germany) at 20× magnification spanning three brain regions namely cortex, subcortex and hippocampus. For each group, a minimum of three animals were analysed from independent experiments. From each animal, three individual brain sections were used for analysis. Per section three images were captured for each brain region, yielding 9 images for each brain region per animal. Processing and quantification of the number and diameter of CD31-immunopositive blood vessels was carried out manually using NIH ImageJ. Vascular density was expressed as vessel number per mm^2^. ZO-1 and claudin-5 staining areas were obtained using the threshold method in NIH ImageJ. Tight junction image analysis was performed by calculating the area of overlap of ZO-1/claudin-5 positive areas with CD31-positive endothelial cells. All quantitative analyses were performed blind.

### Transmission electron microscope (TEM)

Transgenic animals were transcardially perfused using 0.1 M PBS, 2.5% glutaraldehyde, 2% formaldehyde for 15 min then brains were isolated and fixed for 48 h. Cortices were dissected and cut into approximately 1mm^3^ pieces using a vibratome. After washing in 0.1 M CaCo and post fixation in 1% osmium in water on ice for 1 h, tissues were contrasted with 1% uranyl acetate overnight. Following dehydration in an alcohol series brains were embedded in Polypropylene, Propp:Epon-Araldite 1:1 for 1 h, followed by Epon-Araldite for 28 h at 60 °C. Ultrathin sections (70 nm) were cut with a Reichert Ultracut E microtome then post stained with lead for 10 min. Sections imaged using a Phillips CM100 transmission electron microscope with a digital Gatan Orius 832 camera (FEI, The Netherlands).

### ELISA

Measurements of intracellular and serum levels of VEGF and transforming growth factor β (TGF-β) were performed using Quantikine ELISA kits (VEGF: MMV00, TGF-β: DB100B, R&D systems, USA) according to the manufacturer’s instructions using 500 µg of brain protein and fivefold diluted serum. Optical density was measured at 450 nm, with wavelength correction at 570 nm (Multiskan RC; Thermo Labsystems, Finland).

### Statistical analysis

Statistical comparisons were performed using GraphPad Prism 5 software (GraphPad, USA) and all data are expressed as mean ± SD of at least 3 independent experiments. Comparisons among different groups were made using one-way ANOVA with Holm-Sidak post hoc test or two-way ANOVA with Tukey's correction where appropriate. P values < 0.05 were considered significant.

## Results

### Confirmation of perivascular HIF-1 loss of function (LoF)

**Fig. 2:**
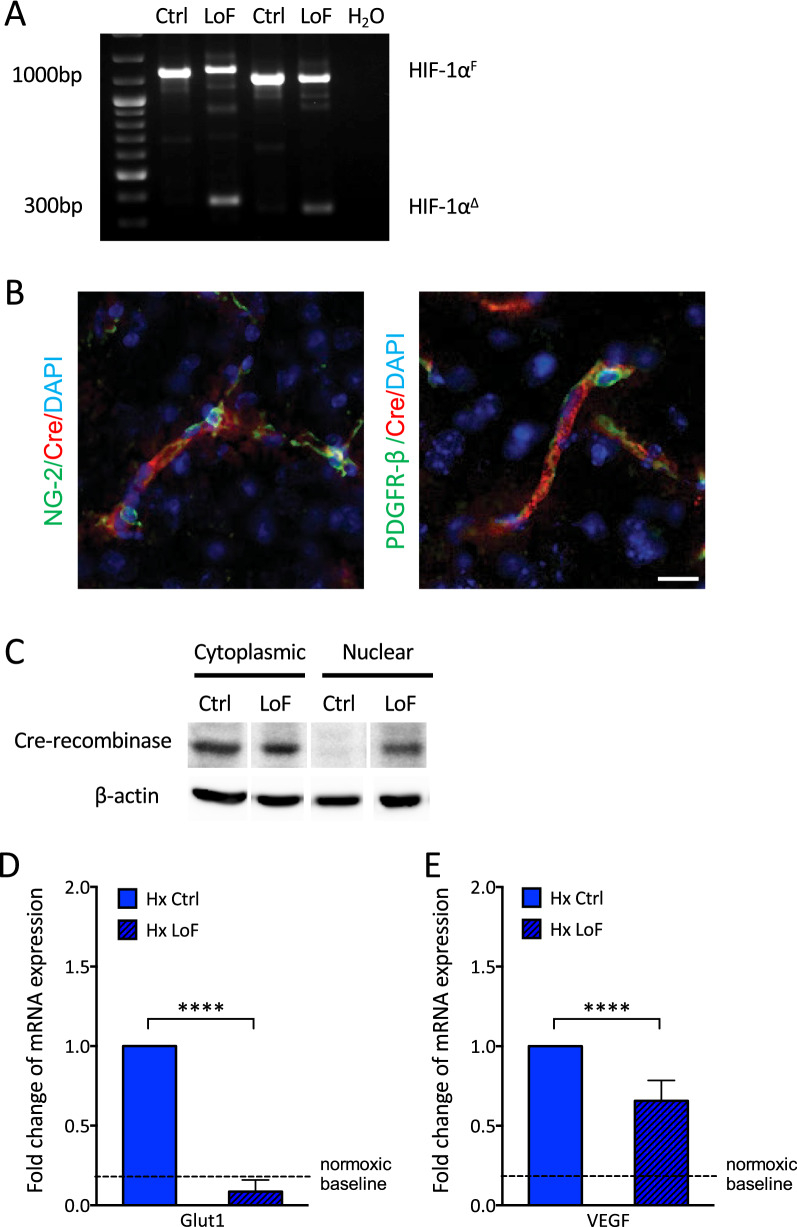
Characterisation of SMMHC-HIF-1α^fl/fl^ mouse line. **A** Genomic PCR genotyping confirming excision of floxed HIF-1α exon 2 in cortical DNA isolated from tamoxifen treated SMMHC-CreER^T2^; HIF-1α^fl/fl^ mice (LoF) in normoxia or hypoxia. A short 300 bp truncated HIF-1α fragment (HIF-1α^Δ^) is produced compared to the 1.1Kbp full length product (HIF-1α^F^) obtained in oil-treated controls. **B** Co-immunostaining with NG-2 or PDGFR-β (green) and Cre recombinase (red) locates Cre recombinase expression specifically to brain pericytes in cortical regions. Scale bar = 50 µm. **C** Immunoblot of brain cytoplasmic and nuclear fractions confirm tamoxifen-induced nuclear translocation of Cre recombinase in SMMHC-CreER^T2^; HIF-1α^fl/fl^ mice. β-actin was used as loading control. Quantitative RT-PCR expression of HIF-1 targets Glut1 (**D**) and VEGF (**E**) in primary pericytes isolated from SMMHC-CreERT2; HIF-1α^fl/fl^ mice and treated with 2 µM tamoxifen (LoF, loss of function) compared to hypoxic vehicle controls (oil, Ctrl) after 48 h hypoxic exposure (1% O_2_). Two-way ANOVA, mean ± SD, n = 4–5. ****p < 0.0001 compared to Hx Ctrl

**Fig. 3:**
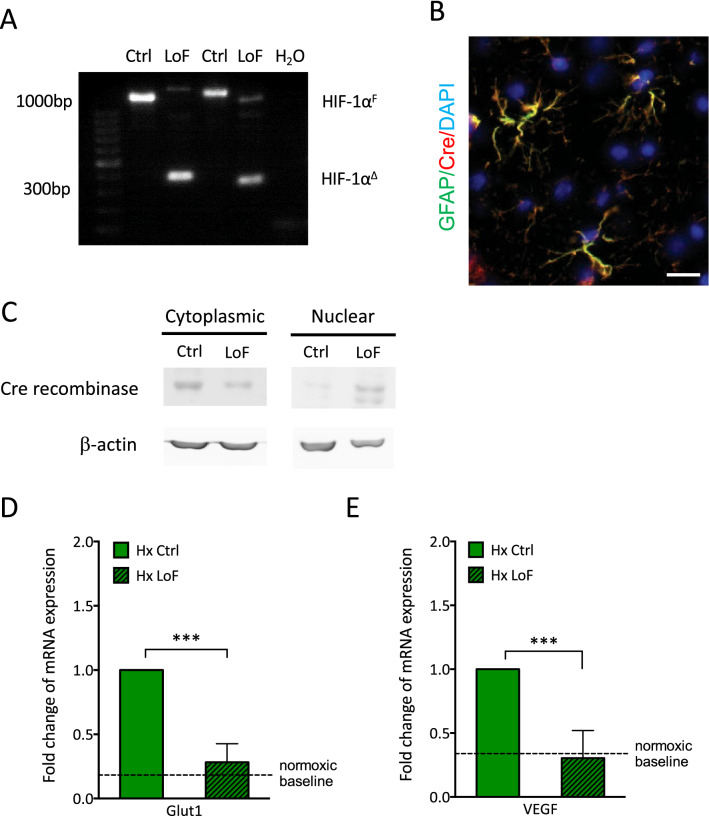
Characterisation of GLAST-HIF-1α^fl/fl^ mouse line. **A** PCR genotyping demonstrates excision of floxed HIF-1α exon 2 in genomic DNA from brain cortices of tamoxifen (LoF) or oil (Ctrl) treated GLAST-CreER^T2^; HIF-1α^fl/fl^ mice. Tamoxifen treatment led to generation of a short 300 bp truncated product of HIF-1α (HIF-1α^Δ^) compared to the 1.1kbp full-length product (HIF-1α^F^). **B** Co-immunostaining of GLAST-CreER^T2^; HIF-1α^fl/fl^ brain sections for GFAP (green) and Cre recombinase (red) in the brain cortex. Scale bar = 50 µm. **C** Immunoblot of brain cytoplasmic and nuclear fractions confirm tamoxifen-induced nuclear translocation of Cre recombinase in GLAST-CreER^T2^; HIF-1α^fl/fl^ mice. β-actin was used as loading control. **D** Glut1 and VEGF mRNA expression in primary astrocytes isolated from GLAST-CreER^T2^; HIF-1α^fl/fl^ mice after tamoxifen (2 µM) or vehicle treatment and 48 h hypoxic exposure (1% O_2_). Two-way ANOVA, mean ± SD, n = 3–5, **p < 0.01, ***p < 0.001 compared to Hx Ctrl

To confirm exon 2 excision after tamoxifen treatment standard genotyping was performed on DNA isolated from cortices of mice using duplex polymerase chain reaction (PCR). In all lines only the WT allele (HIF-1α^F^, 1000 bp) was detected after oil treatment whereas both WT and exon 2 floxed fragments (HIF-1α^Δ^, 300 bp) were amplified after tamoxifen treatment as shown in SMMHC-LoF (Fig. 2A) and GLAST-LoF (Fig. 2A) mice. This outcome was unaffected by hypoxic exposure. Immunofluorescence staining showed that Cre expression co-localizes with PDGFR-β and NG2 in the SMMHC line (Fig. 2B), and GFAP-positive vascular-associated cells in the GLAST line as expected (Fig. 3B). Western blot analysis  confirmed tamoxifen treatment induces translocation of Cre from cytoplasmic to nuclear cortical fractions in the different CreER^T2^; HIF-1α^fl/fl^ mouse lines confirming recombinase activation (Figs. 2C and 3C and Additional file 1: Fig. S2). Finally, loss of HIF-1 activity and function was verified by evaluating hypoxic induction of its target genes in the cellular specific compartments. To this end primary astrocytes and pericytes were isolated and cultured from the mice then exposed to either tamoxifen or oil prior to normoxic or hypoxic exposure. Quantitative real-time PCR showed mRNA levels of key HIF-1 target genes Glut1 (Figs. 2D and 3D) and VEGF (Figs. 2E and 3E) were induced by hypoxia in both oil treated pericytes and astrocytes but significantly abrogated in tamoxifen exposed cells as expected.

### Pericyte- but not astrocyte-targeted HIF-1-LoF prevents hypoxia-mediated vessel dilation

**Fig. 4:**
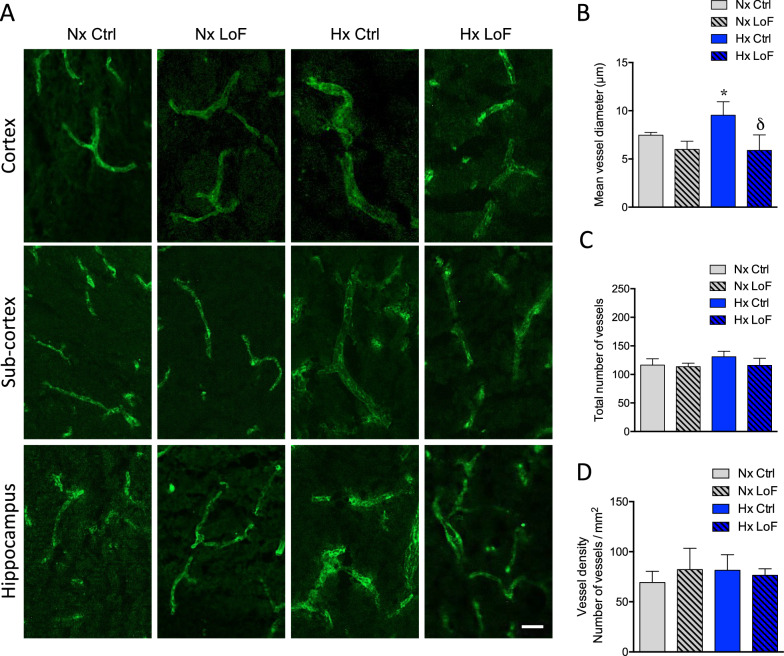
Pericyte-targeted HIF-1 deletion prevents hypoxia-induced vessel dilation. **A** Representative images of brain microvessels stained with CD31 (green) in cortex, subcortex and hippocampal regions of SMMHC-CreER^T2^; HIF-1α^fl/fl^ mice treated with oil (Ctrl) or tamoxifen (LoF) after 96 h normoxia (Nx, 21% O_2_) or hypoxia (Hx, 8% O_2_). Scale bar = 50 µm. Quantification of mean vessel diameter (**B**), number of vessels (**C**) and vessel density (**D**) in brain cortical regions during normoxia and hypoxia in Ctrl and LoF mice. Two-way ANOVA, mean ± SD, n = 3, *p < 0.05, **p < 0.01, compared to Nx Ctrl, δp < 0.05, compared to Hx Ctrl

Sustained exposure to hypoxia is known to induce endothelial remodelling in different disease paradigms [40] but whether perivascular HIF-1 stabilization influences microvascular stability still needs to be addressed. We performed immunostaining with an anti-CD31 antibody to evaluate if HIF-1 LoF in pericytes or astrocytes modulates vessel characteristics when mice are exposed to 21% or 8% O_2_ for 96 h. As expected brain sections from normoxic Ctrl and LoF SMMHC-Ctrl mice showed no apparent differences in vessel structure (Fig. 4A). A clear hypoxia-induced increase in vessel diameter in all regions including cortex, sub-cortex and hippocampus was noted in the hypoxic SMMHC-Ctrl mice whereas, surprisingly, vessels of hypoxic SMMHC-LoF mice looked very similar to normoxic controls (Fig. 4A). Quantitative analysis of mean vessel diameter confirmed hypoxia induced vessel dilation was abrogated in cortex (Fig. 4B) as well as subcortex and hippocampus (Additional file 1: Fig. S3A, B). Notably, total number of cortical vessels and vessel density was largely unaffected (Fig. 4C, D).

**Fig. 5:**
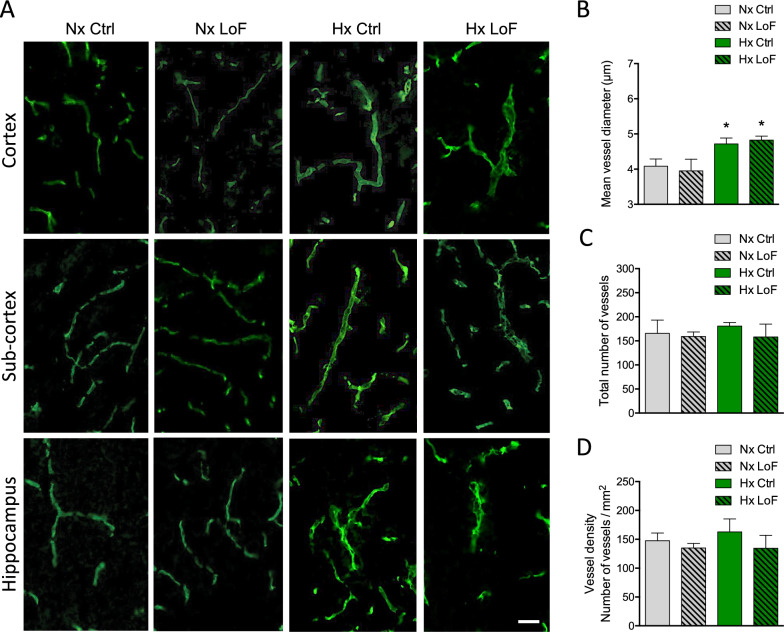
No effect of astrocyte HIF-1 LoF on vascular characteristics. **A** Representative images of CD31-stained microvessels (green) in cortex, sub-cortex and hippocampus of GLAST-CreER^T2^;HIF-1α^fl/fl^ mice treated with or without tamoxifen after 96 h normoxic or hypoxic exposure. Scale bar = 50 µm. Quantification of cortical mean vessel diameter (**B**), vessel number (**C**) and vessel density (**D**). Two-way ANOVA, mean ± SD, n = 3, *p < 0.05 compared to Nx Ctrl and Nx LoF

HIF-1 LoF also had no effect on normoxic vessel diameter in the GLAST (Fig. 5A) or GFAP astrocyte lines (data not shown) and did not abrogate the hypoxia-mediated vessel dilation seen in the Ctrl animals (Fig. 5A). Quantification confirmed increased mean vessel diameter in cortex (Fig. 5B) as well as subcortex and hippocampal (Additional file 1: Fig. S3C, D) regions of hypoxic GLAST mice. Similar data was obtained using GFAP LoF mice (Additional file 1: Fig. S4A–C). Notably however, HIF-1 LoF had no effect as both the total number of vessels and vessel density remained constant (Fig. 5C, D). Thus pericyte, but not astrocyte, HIF-1 LoF prevents hypoxia-mediated vessel dilation.

### Pericyte HIF-1 LoF prevents hypoxia-induced brain vascular permeability

**Fig. 6:**
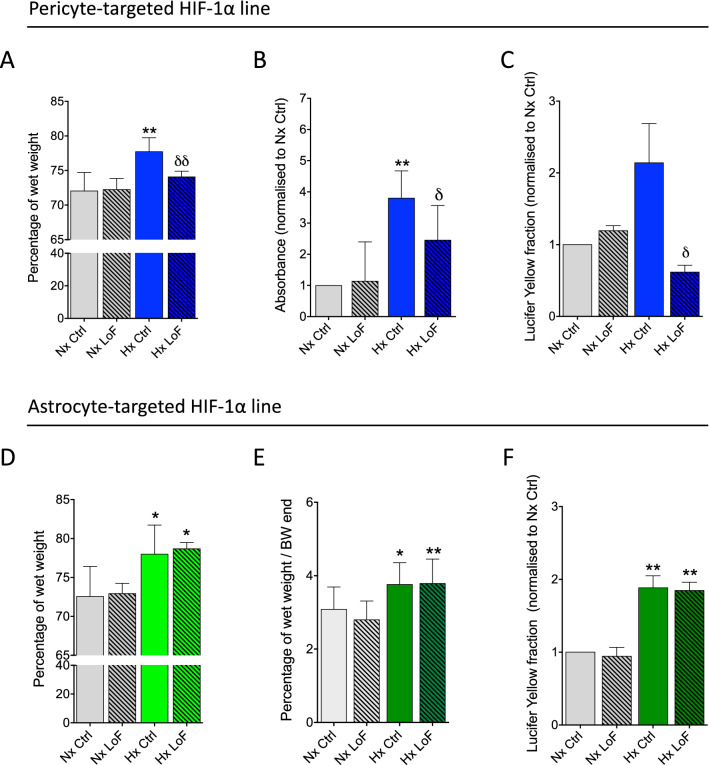
Pericyte- but not astrocyte HIF-1 LoF modulates barrier permeability during hypoxia. Hypoxia-induced changes in brain vascular permeability as measured by **A** brain water content, **B** Evans blue and **C** Lucifer Yellow dye extravasation into brain tissue of SMMHC-CreER^T2^; HIF-1α^fl/fl^ mice treated with oil (Ctrl) or tamoxifen (LoF) after 96 h normoxia (Nx, 21% O_2_) or hypoxia (Hx, 8% O_2_). Brain water content changes in (**D**) GFAP-CreER^T2^; HIF-1α^fl/fl^ and (E) GLAST-CreER^T2^; HIF-1α^fl/fl^ control or LoF mice after normoxic or hypoxic exposure. (F) Lucifer Yellow dye extravasation into brain tissue of GLAST-CreER^T2^; HIF-1α^fl/fl^ mice. Two-way ANOVA, mean ± SD, n = 5 -8, *p < 0.05, **p < 0.01, compared to Nx Ctrl and Nx LoF, δp < 0.05, δδp < 0.01 compared to Hx Ctrl

Hypoxia-driven vascular remodelling is highly associated with increased vascular permeability in vivo [40]. Unexpectedly, the absence of detectable vascular modifications in both the GLAST and GFAP lines implied little contribution of astrocyte activated HIF-1 pathways to hypoxia-induced vascular changes. In contrast, since blocking pericyte-mediated HIF-1 signalling suppressed vessel dilation it seemed highly likely that BBB integrity was also better maintained in these animals. To directly evaluate barrier functionality we assessed vascular permeability using different functional assays. First, we quantified brain edema by measuring water content (i.e. % wet weight) of whole brain tissue after 96 h normoxic or hypoxic exposure. Significantly increased wet weight in SMMHC-Ctrl mice during hypoxic conditions were appreciably abrogated in the SMMHC-LoF animals (Fig. 6A)*.* In correlation functional permeability assays showed hypoxia substantially increased paracellular flux of both Evans blue (Fig. 6B) and Lucifer yellow (Fig. 6C) tracers in SMMHC-Ctrl animals as expected, but in both cases this effect was abrogated in SMMHC-LoF animals. In complete contrast, and in correlation with the inability of astrocyte HIF-1-LoF to suppress hypoxia-induced vascular dilation, endothelial permeability remained unaffected. Indeed, water content as well as Lucifer yellow and Evans blue flux was constant in both hypoxic GFAP- and GLAST-LoF mice compared to controls (Fig. 6D–F). Thus specifically reducing pericyte but not astrocyte HIF-1 activation suppresses hypoxia-induced BBB permeability. Of note, no permeability changes were measured when analysing the tracer dye Texas Red (Additional file 1: Fig. S5A, B).

### Pericyte HIF-1 deletion prevents hypoxic barrier disruption without altering key vascular modulatory signalling pathways

**Fig. 7:**
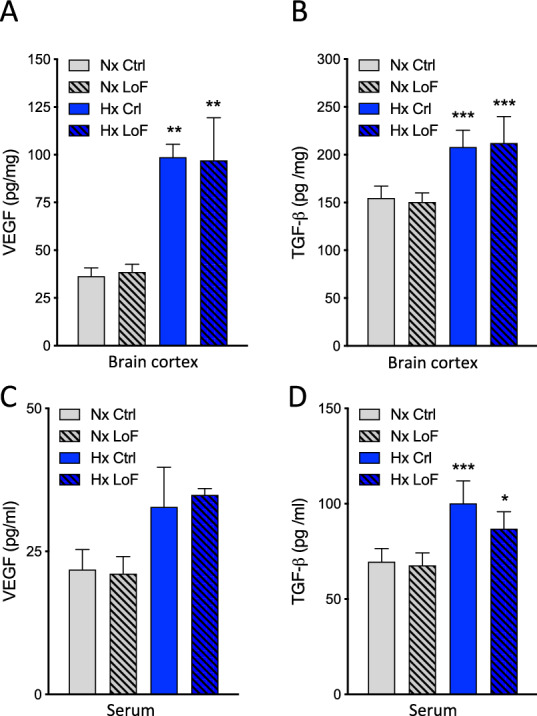
HIF-1 deletion prevents hypoxic barrier disruption without altering VEGF or TGF-β levels in brain tissue or serum. VEGF and TGF-β levels measured by ELISA in brain cortex protein lysates (pg/mg normalized to total protein content, panels **A**, **B**) and serum (pg/ml, panels **C**, **D**) of SMMHC-CreER^T2^; HIF-1α^fl/fl^ mice treated with (LoF) or without (Ctrl) tamoxifen after 96 h normoxia (Nx, 21% O_2_) or hypoxia (Hx, 8% O_2_). Two-way ANOVA, mean ± SD, n = 3 **p < 0.01 compared to Nx Ctrl. Two-way ANOVA, mean ± SD, n = 5, *p < 0.05, **p < 0.01, ***p < 0.001 compared to Nx Ctrl and Nx LoF

Since hypoxia-induced vascular alterations were resolved only in SMMHC-LoF animals we focused on this line in further experiments. To understand how BBB permeability was modulated we first assessed if HIF-1 LoF alters pericyte survival or proliferation. Only few Ki-67 positive pericytes were observed after hypoxia with no difference observed between Ctrl and LoF mice and no proliferation of other cells was detected (Additional file 1: Fig. S6A). Similarly no cell death (measured by TUNEL) was observed at all (Additional file 1: Fig. S6B) suggesting that hypoxic BBB integrity is primarily modulated by HIF-1-driven alterations in pericyte signalling. Several downstream mediators are potential candidates for the mechanism. We focused on VEGF, TGF-β and MMPs as they are HIF-1 targets known to be strong mediators of vascular permeability that can be secreted and/or activate cognate receptors on the endothelium [41]. Analyses of both brain protein lysates and serum samples by ELISA showed increased levels of VEGF and TGF-β during hypoxia as expected but no alterations after pericyte HIF-1 LoF (Fig. 7A-D). No changes in MMP-2 or 9 by either zymography or in situ zymography were detected (data not shown).

### Hypoxia-induced TJ disruption is rescued by pericyte-targeted HIF-1 deletion

**Fig. 8:**
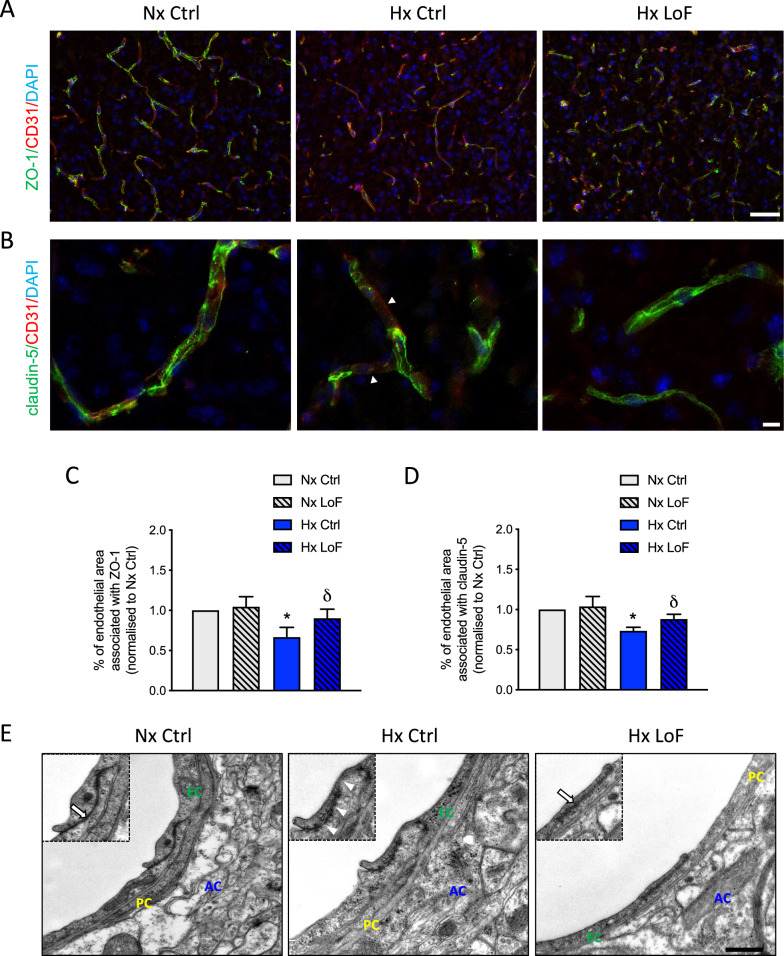
Hypoxia-induced TJ disruption is rescued by pericyte-targeted HIF-1 deletion. Representative images of SMMHC-CreER^T2^; HIF-1α^fl/fl^ Ctrl and LoF brain sections co-immunostained for junctional proteins (**A**) ZO-1 or (**B**) claudin-5 (green, Z-stack images) with CD31 (red), from mice exposed to 96 h normoxia (Nx) and hypoxia (Hx, 8% O_2_). Nuclei are counterstained with DAPI (blue). Arrowheads highlight loss of TJ staining within microvessels. Scale bars = 100 µm and 25 µm, respectively. Quantification of ZO-1 (**C**) and claudin-5 (**D**) staining in brain cortical regions of HIF-1 Ctrl and LoF mice during normoxia and hypoxia. Two-way ANOVA, mean ± SD, n = 3–4, *p < 0.05 compared to Nx Ctrl, δp < 0.05 compared to Hx Ctrl. **E** Representative microvessel electron microscopy images from SMMHC-CreER^T2^; HIF-1α^fl/fl^ mice exposed to different conditions showing tight junction arrangement between adjacent endothelial cells. Astrocytes (AC) are indicated in blue font, pericytes (PC) in yellow and endothelial cells (EC) in green font. Insets highlight intact (arrow) or disrupted (arrowhead) TJ structures at endothelial cell borders. Scale bar = 0.5 µm

Finally we assessed if microvessel tight junction organization was altered by pericyte-targeted HIF-1 LoF. Immunostaining of brain sections from normoxic and hypoxic SMMHC-Ctrl and SMMHC-LoF mice was performed for the tight junction proteins occludin, claudin-5 and ZO-1 (Fig. 8) in combination with the blood vessel marker CD31. Low power ZO-1 images (Fig. 8A) provide an overview of vessel structure and regional TJ expression whereas high power claudin-5 images (Fig. 8B) highlight TJ organization. In control animals (Hx Ctrl) an evident reduction of vessel localized ZO-1 staining was observed with regions of complete loss of signal compared to Nx Ctrls. Although similar disruption of the vessels was seen in Hx LoF mice, ZO-1 expression was more similar to normoxic animals with reduced disrupted or discontinuous staining. High power Z-stack images of claudin-5 staining (green, Fig. 8B) revealed multiple breaks and regions of complete signal loss at vessel walls in hypoxic SMMHC-Ctrl animals (Hx Ctrl) as indicated by arrow heads. In contrast hypoxic SMMHC-LoF mice (Hx LoF) showed considerably improved TJ localization that was more aligned to normoxic animals without disrupted or discontinuous staining. Comparable results were obtained for occludin (data not shown). Quantification of the staining further confirmed that hypoxia-induced ZO-1 and claudin-5 disruption is rescued in Hx SMMHC-LoF mice (Fig. 8C, D). To assess TJ arrangement in even more detail, direct transmission electron microscopy was performed on brain sections from these animals. Results confirmed that pericyte HIF-1 LoF improves tight junction organization during hypoxia (Fig. 8E). Close apposition of two endothelial membranes is apparent in normoxic SMMHC-Ctrl (Nx Ctrl) as indicated by the arrow. This organization is compromised during hypoxia (Hx Ctrl) wherein the uniform pattern of electron dense particles at the TJ becomes more akin to a string of pearls and gaps are clearly evident (arrowheads, Fig. 8E). In contrast hypoxic SMMHC-LoF animals (Hx LoF) display tight junction arrangement very similar to normoxic animals.

## Discussion

The brain perivascular compartment is well known to play an important role in modulating barrier functionality physiologically but key mechanisms that underlie their responses to pathological insults such as hypoxia or ischemia, and subsequent impact on barrier integrity are considerably less well defined. At the cellular level, many injury-induced outcomes are closely entwined with activation of the HIF-1 signalling pathway as it enables cells to rapidly adapt to environmental change. On the flip side however, HIF-1 stabilization can also activate apoptotic pathways or other mechanisms that facilitate cell death. Although in vitro and in vivo evidence continues to advocate that HIF-1 signalling increases barrier permeability [20–27], the contribution of perivascular cells to this outcome is unclear. Using sole oxygen deprivation, i.e. hypoxia, we asked whether astrocyte and/or pericyte stabilization of HIF-1 plays a pivotal role in injury-induced vascular modulation. Our data shows pericyte HIF-1 induction is highly consequent to vascular permeability and stability whereas astrocyte HIF-1 stabilization has no obvious impact.

Astrocytes are well known to store and release large amounts of permeability inducing factors during injury conditions [11, 42]. The strong correlation between secretion of such factors and vascular dysfunction seems to have led to the forgone conclusion that astrocytes are the culprits for barrier disturbance. On closer look at many reports however, we note that these conclusions have been drawn largely from correlative studies and direct proof is very much lacking. Herein, we show using two mouse models (GFAP and GLAST) that blocking astrocyte HIF-1 function has no measurable impact on VEGF or TGF-β levels, the hypoxic vascular response or indeed BBB stability. Although surprising, this outcome agrees with the observation that during severe inflammatory conditions astrocyte VEGF secretion was directly related to injury outcome, but independent of HIF-1 [11]. Similarly, we recently showed that media from hypoxic astrocytes with HIF-1 deletion also had no impact on endothelial characteristics or VEGF levels in vitro [43]. A number of possibilities could underlie these observations. First, the molecular stores released by astrocytes physiologically might be different to those that induce permeability during injury processes. In the case of VEGF and TGF-β this would align with the fact that many isoforms have different functionality and are differentially expressed by different cell types [44, 45]. Although the possibility of differences in physiological versus pathological secreted VEGF isoforms (or their targets) has not been investigated, it is important to note that astrocytes constantly secrete VEGF during development and adulthood under physiological conditions [46, 47] and in the absence of detectable HIF-1 levels [11]. Another option, perhaps frequently overlooked, is that directionality of the signals might be vital i.e. not all injury-induced secreted molecules necessarily target the endothelium. Indeed cellular organization at the NVU means astrocytes communicate directly with neurons and pericytes as opposed to largely indirectly with endothelial cells. Evidence for, or against, this argument is lacking but it clearly deserves more investigation. Despite not being able to discount a different outcome during harsher scenarios or more chronic exposure times, generally our data suggests that astrocyte HIF-1 stabilization plays a role that likely excludes vascular modulation. We speculate that cytoprotective autocrine and paracrine effects are decisive as suggested by others [48, 49]. For example, endogenous VEGF secretion during injury increased astrocyte survival and proliferation per se*,* as well as provided neuroprotection [48, 50]. Our data clearly suggests pericyte HIF-1 is more relevant for vascular integrity.

Sharing a basement membrane with endothelial cells means pericytes have high potential to control endothelial function, and thus BBB stability, under all conditions. A very recent study highlighted this intimate involvement by showing pericytes and endothelial cells form a functional relay unit by coupling to each other but not to surrounding neurons and glia [51]. Since pericyte HIF-1 LoF considerably improved microvascular stability it seems clear that pathway activation in these cells promotes vascular disturbance. This agrees with another study from our group where deletion of pericyte HIF-1 also improved outcome after stroke [52]. The mechanism(s) behind these important findings however remains unclear.

Pericyte tolerance per se significantly impacts cell–cell communication and BBB stability during injury [53–55]. Although pericytes are more susceptible to injury compared to astrocytes in vitro [37, 55] and also exhibit negative responses in vivo [56–58], we did not observe any changes in pericyte number or localization in this study. This contrasts with recently published data showing pericyte death and/or movement away from vessel walls during stroke has detrimental outcome [59], as well as our own study showing HIF-1 mediated pericyte death during transient brain ischemia leads to barrier dysfunction and compromised recovery [52]. However, we must take into account that 96 h hypoxic exposure at 8% O_2_ is a less severe/acute injury than stroke, and one that the animals tolerate reasonably well. Accordinlgy, wide-spread cell death is unlikely to occur. As 96 h exposure at 8% was selected to enable consistent measurement of barrier compromise without dramatically impacting well-being of the mice, we cannot discount that pericytes might mediate BBB permeability at earlier time points. Absence of pericyte alterations per se post hypoxic challenge suggests that altered HIF-1 downstream signalling is more likely to be important. It was therefore intriguing that similarly to the astrocyte lines we did not detect significant changes in key HIF-1-induced molecules known to impact BBB stability such as VEGF, MMPs or TGF-β as suggested by others [11, 27, 60, 61]. However, as there are more than 300 HIF-1 target genes the possibility of direct and/or indirect effects by numerous other candidates seems endless. We also remain mindful that small local changes in signalling within the vessel compartment are not easily detected in brain tissue lysates in vivo*.* We thus hope that on-going vessel isolation experiments will provide better insight. Recent evidence suggests that gap junction-mediated coupling between endothelial cells and pericytes is critical for vascular functionality [51]. How disrupted connexin signalling results in severe non-brain vascular dysfunction was recently reviewed [62], but a link between HIF-1 and connexins and/or disturbed gap junction communication signalling has to our knowledge not yet been shown.

It is noteworthy that hypoxia can stimulate pericytes to switch from a quiescent to active or even pro-angiogenic state [58]. This seems to be a pericyte-specific outcome as other mural cells, smooth muscle cells, responded oppositely [58]. Interestingly, the authors also observed a similar hypoxia-induced activation in endothelial cells suggesting an overall hypoxic microvascular response. This may again tie back to cell-specific susceptibility to hypoxia in general and/or molecular and metabolic reprogramming. As stimulating pericyte glycolysis causes them to exit the quiescent state [63] and HIF-1 strongly regulates the glycolysis pathway, it is highly feasible that hypoxia-induced HIF-1 stabilisation culminates in pericyte activation whereas HIF-1 LoF supports pericyte quiescence. Considering such responses are entwined with vessel functionality, more investigation of how pericyte HIF-1 regulates the vascular compartment is clearly needed.

## Conclusion

HIF-1 stabilization in pericytes, but not astrocytes, underlies hypoxic vascular dysfunction. Our data clearly emphasizes the importance and complexity of cell-specific autocrine and paracrine signalling within the neurovascular compartment. Only by understanding the mechanisms at work in considerably more detail will we get a handle on how to modulate barrier functionality during injury and disease progression.

## Supplementary Information

**Additional file 1: Figure S1.** Characterization of the transgenic mouse lines. (A) Flowchart of experimental setup including tamoxifen injection, exposure duration and endpoint measurements. (B) Hematocrit levels in SMMHC-CreER^T2^; HIF-1α^fl/fl^ (blue), GFAP-CreER^T2^; HIF-1α^fl/fl^ (light green) and GLAST-CreER^T2^;HIF-1α^fl/fl^ (dark green) mice treated with oil (Ctrl) or tamoxifen (LoF) after exposure to 96 h normoxia (Nx, 21% O_2_) or hypoxia (Hx, 8% O_2_). Two-way ANOVA, mean ± SD, n = 3–8, ****p < 0.0001 compared to Nx Ctrl and Nx LoF. **Figure S2.** Raw blots confirming Cre recombinase nuclear translocation in SMMHC-CreER^T2^; HIF-1α^fl/fl^ mice and GLAST-CreER^T2^; HIF-1α^fl/fl^ mice. (A) Cytoplasmic and nuclear protein fractions were extracted from brain tissue of an oil treated animal (Oil) or mice injected with increasing concentrations of tamoxifen (25, 50 or 125 mg/kg body weight) for 5 days. Positive (C1 & C2) and negative controls (neg) were included. β-actin was used as loading control after subsequent stripping of the membrane. Lanes with the tamoxifen concentration used throughout this study and as presented in Fig. 2A are boxed in red. (B) Nuclear and cytoplasmic protein fractions were extracted from brain tissue of two oil (Ctrl) or tamoxifen (LoF) treated animals. β-actin was used as loading control after subsequent stripping of the membrane. Lanes presented in Fig. 3A are boxed in red. **Figure S3.** Vessel diameter changes in SMMHC and GLAST transgenic mice. Quantification of mean vessel diameter in subcortex (A, C) and hippocampus (B, D) of SMMHC- and GLAST-CreER^T2^; HIF-1α^fl/fl^ mice respectively, treated with oil (Ctrl) or tamoxifen (LoF) and exposed to normoxia or hypoxia (Hx, 8% O_2_) for 96 h. Two-way ANOVA, mean ± SD, n = 3, *p < 0.05 compared to Nx Ctrl. **Figure S4. **Vessel diameter changes in GFAP-CreER^T2^;HIF-1α^fl/fl^ mice. Mean vessel diameter in (A) cortex, (B) subcortex and (C) hippocampus regions of GFAP-CreER^T2^;HIF-1α^fl/fl^ mice treated with oil (Ctrl) or tamoxifen (LoF) and exposed to normoxia or hypoxia (Hx, 8% O_2_) for 96 h are presented. Mean ± SD, n = 2. **Figure S5.** Barrier permeability Texas Red dextran is unaltered. Texas Red dextran dye (70 kDa) extravasation into brain tissue of SMMHC-CreER^T2^; HIF-1α^fl/fl^ (A) or GLAST-CreER^T2^;HIF-1α^fl/fl^ (B) mice treated with oil (Ctrl) or tamoxifen (LoF) after 96 h normoxia (Nx, 21% O_2_) or hypoxia (Hx, 8% O_2_). Mean ± SD, n = 3–5. **Figure S6.** No endothelial cell proliferation or apoptosis in SMMHC-CreER^T2^;HIF-1α^fl/fl^ mice. (A) Representative images evaluating cell proliferation in brain sections of SMMHC-CreER^T2^;HIF-1α^fl/fl^ treated with oil (Ctrl) or tamoxifen (LoF) and exposed to hypoxia (Hx, 8% O_2_). Sections were labelled with the proliferation marker Ki-67 (green) and the endothelial marker tomato lectin (red), nuclei were counterstained with DAPI (blue). Scale bar = 200 µm. (B) Representative images evaluating endothelial cell apoptosis in SMMHC-CreER^T2^;HIF-1α^fl/fl^ mice. Co-immunofluorescent labelling of brain sections for TUNEL (green) and CD31 (red), with DAPI counterstain (blue) is shown. n = 3. Scale bar = 200 µm.

## Data Availability

The datasets used and/or analysed during the current study are available from the corresponding author on reasonable request.

## Abbreviations

BBB: Blood–brain barrier
CD31: Cluster of differentiation 31
CNS: Central nervous system
GFAP: Glial fibrillary acidic protein
GLAST: Glutamate aspartate transporter
Glut1: Glucose transporter 1
HIF-1: Hypoxia-inducible factor 1
LoF: Loss of function
MMP: Matrix metalloproteinase
NG-2: Neural glial antigen 2
NVU: Neurovascular unit
PDGFRβ: Platelet-derived growth factor β
SMMHC: Smooth muscle myosin heavy chain
TEM: Transmission electron microscopy
TGF-β: Transforming growth factor β
TJ: Tight junction
TUNEL: Terminal deoxynucleotidyl transferase dUTP nick end labeling
VEGF: Vascular endothelial growth factor
ZO-1: Zonula occludens 1
