# Supplementary figures and images for: Pericyte, but not astrocyte, hypoxia inducible factor-1 (HIF-1) drives hypoxia-induced vascular permeability in vivo
Source: Fluids Barriers CNS. 2022 Jan 15;19:6. doi: 10.1186/s12987-021-00302-y (PMC8760662; doi:10.1186/s12987-021-00302-y)

Suppl. Figure 1

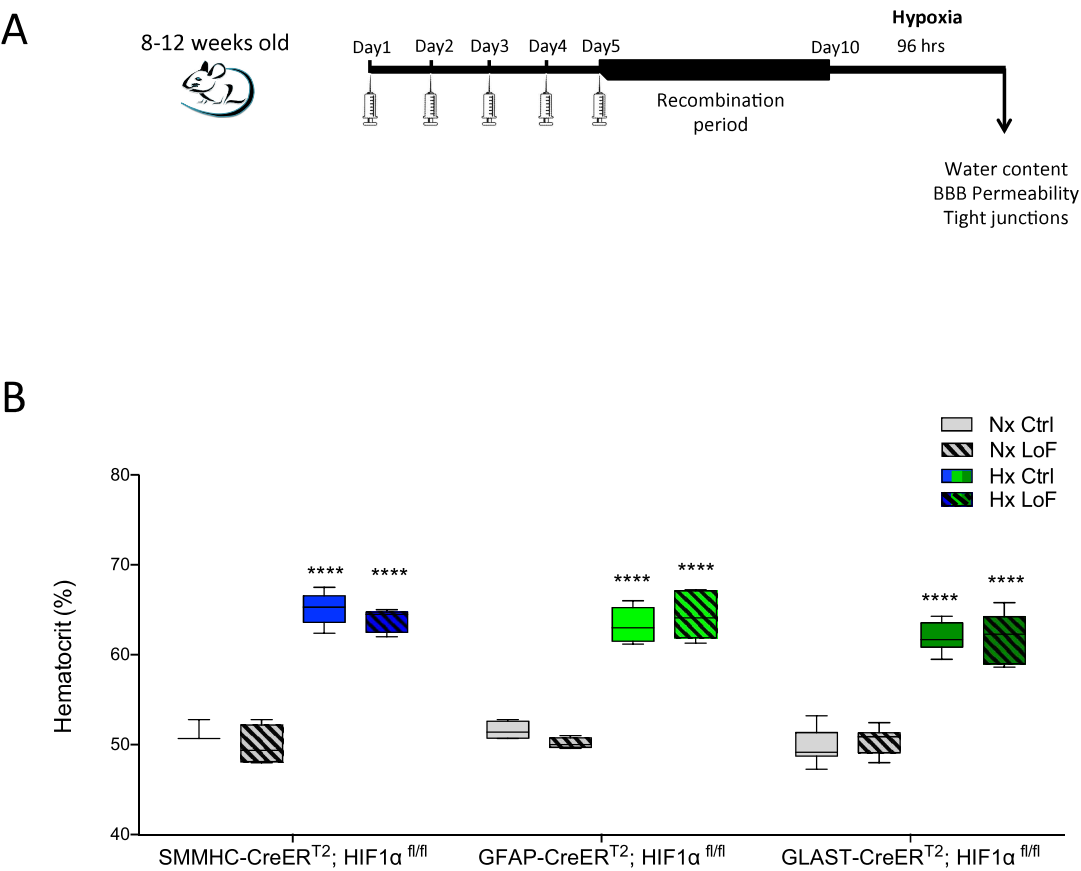

Suppl. Figure 2

A

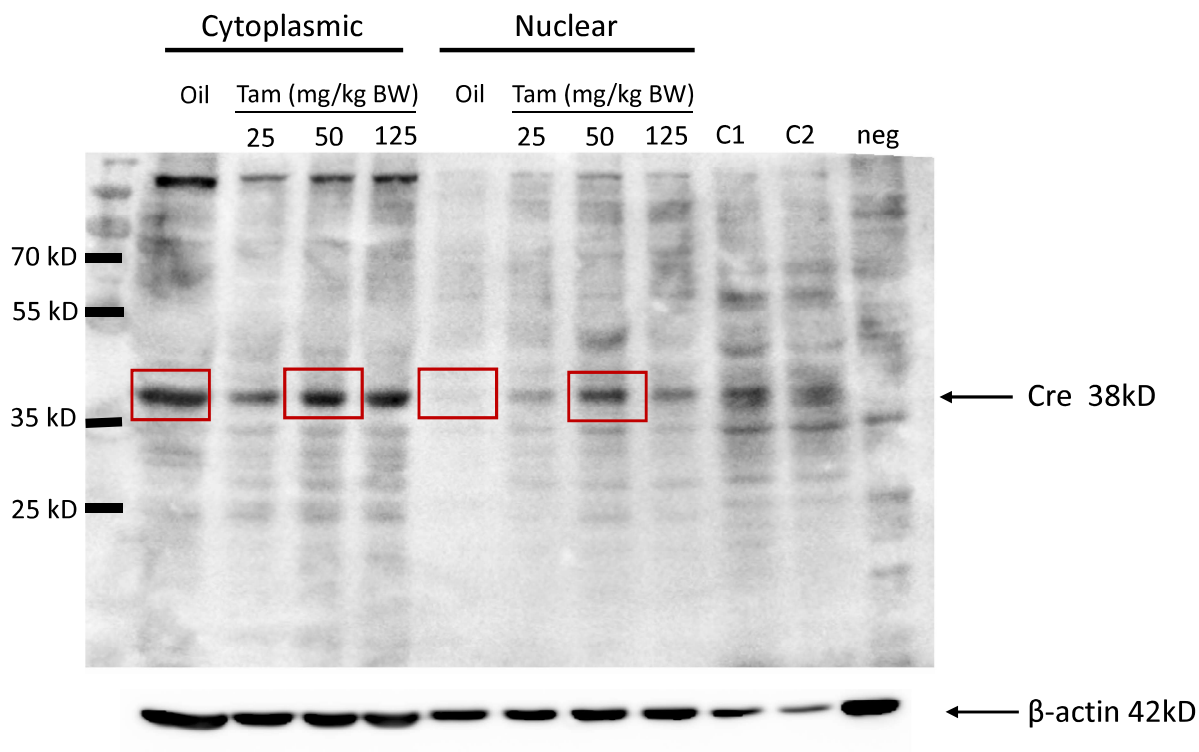

B

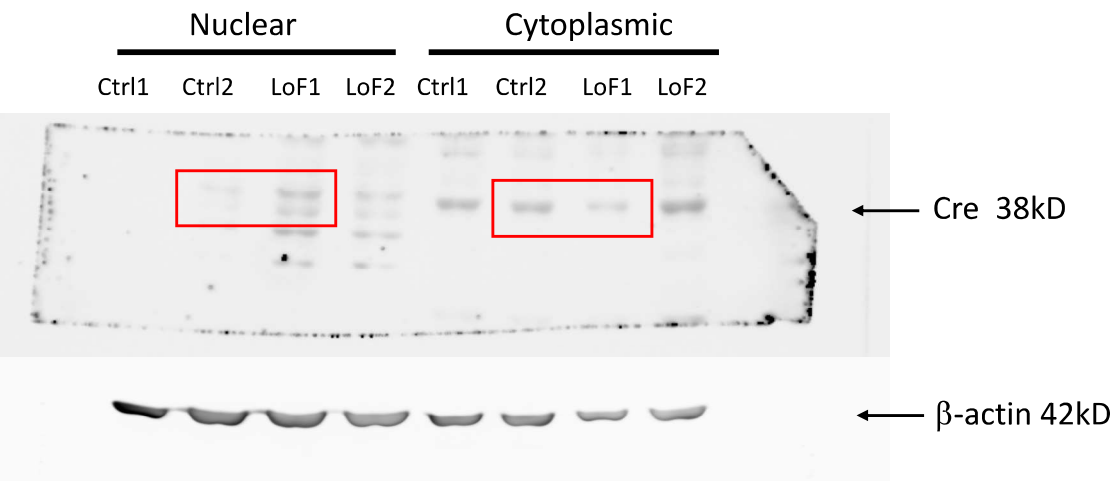

Suppl. Figure 3

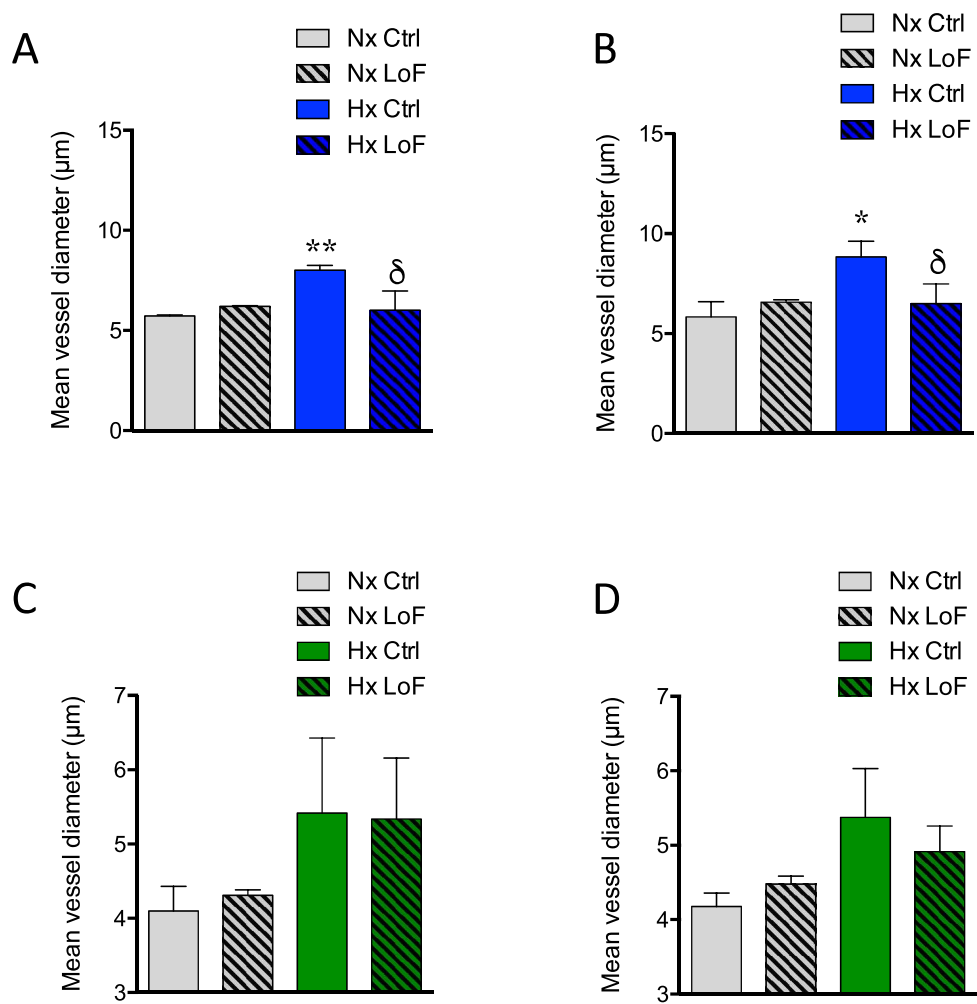

Suppl. Figure 4

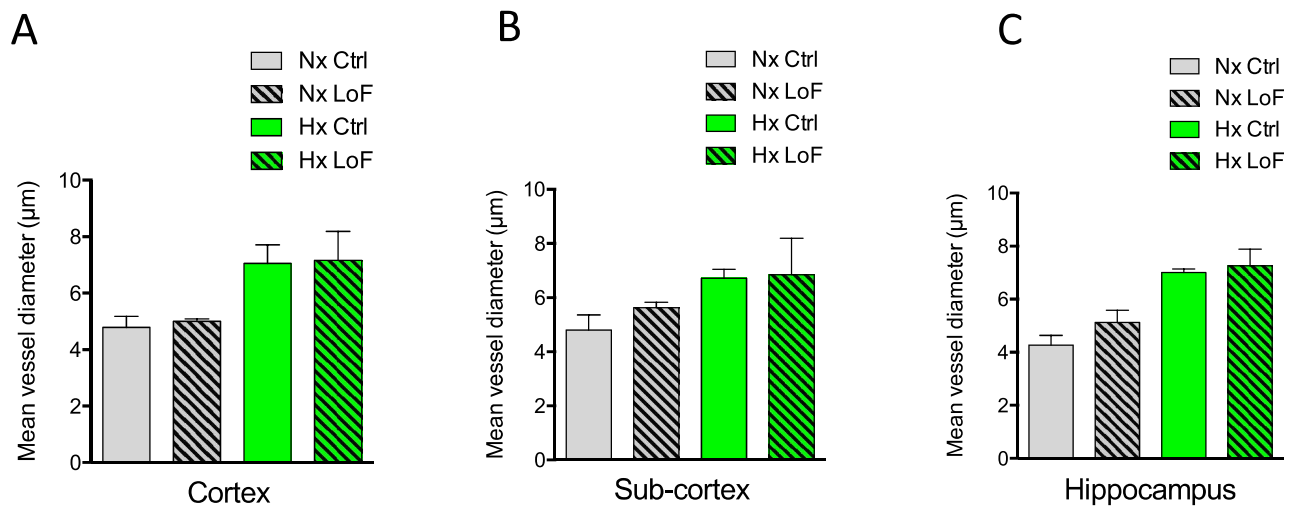

Suppl. Figure 5

**A**

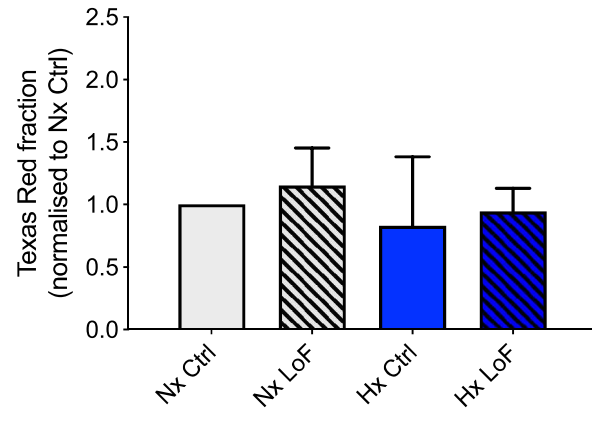

**B**

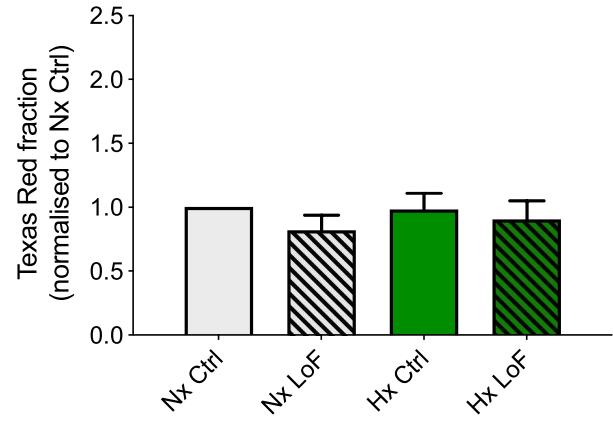

Suppl. Figure 6

A

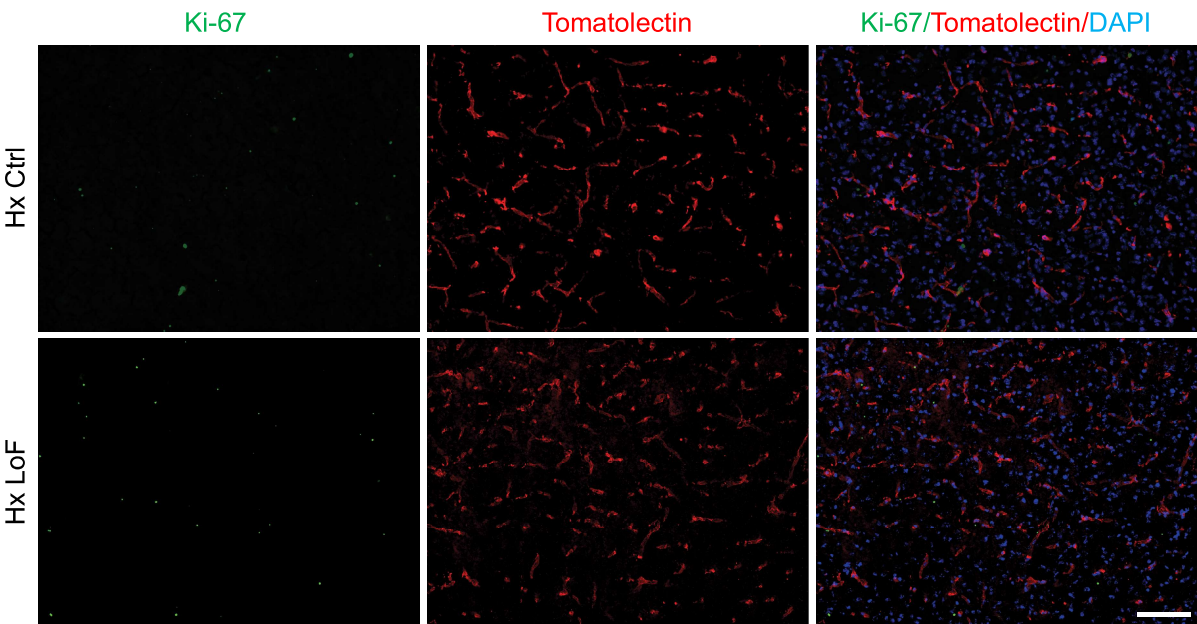

B

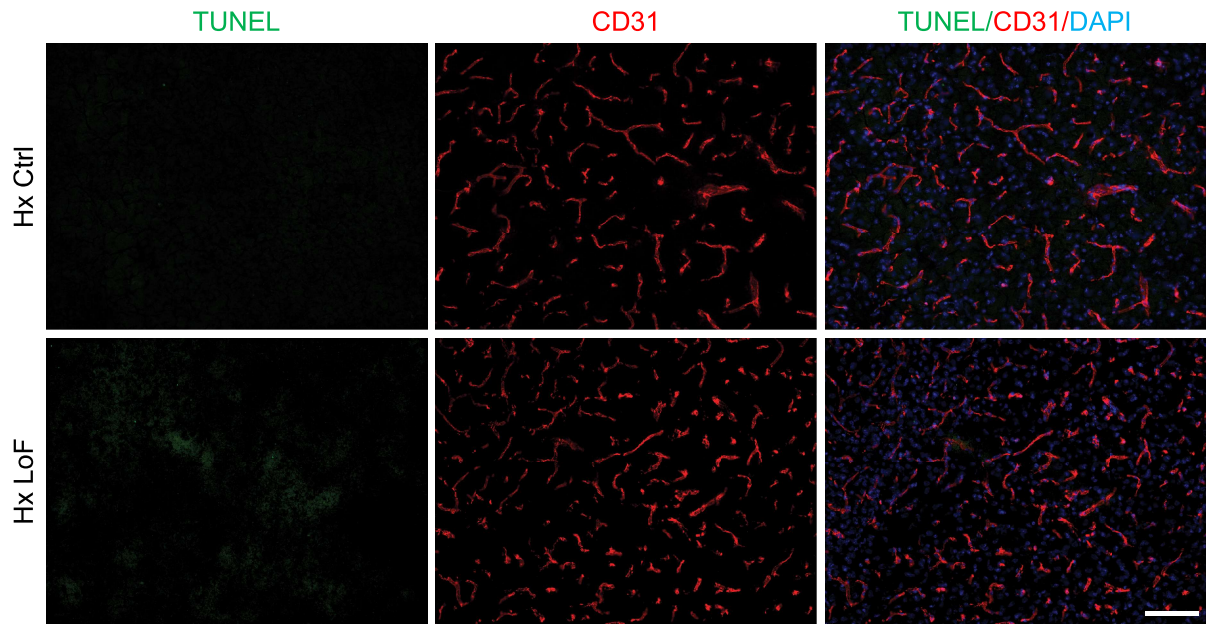

Supplement: Supplementary file 1 — Additional file 1: Figure S1. Characterization of the transgenic mouse lines. (A) Flowchart of experimental setup including tamoxifen injection, exposure duration and endpoint measurements. (B) Hematocrit levels in SMMHC-CreERT2; HIF-1αfl/fl (blue), GFAP-CreERT2; HIF-1αfl/fl (light green) and GLAST-CreERT2;HIF-1αfl/fl (dark green) mice treated with oil (Ctrl) or tamoxifen (LoF) after exposure to 96 h normoxia (Nx, 21% O2) or hypoxia (Hx, 8% O2). Two-way ANOVA, mean ± SD, n = 3–8, ****p < 0.0001 compared to Nx Ctrl and Nx LoF. Figure S2. Raw blots confirming Cre recombinase nuclear translocation in SMMHC-CreERT2; HIF-1αfl/fl mice and GLAST-CreERT2; HIF-1αfl/fl mice. (A) Cytoplasmic and nuclear protein fractions were extracted from brain tissue of an oil treated animal (Oil) or mice injected with increasing concentrations of tamoxifen (25, 50 or 125 mg/kg body weight) for 5 days. Positive (C1 & C2) and negative controls (neg) were included. β-actin was used as loading control after subsequent stripping of the membrane. Lanes with the tamoxifen concentration used throughout this study and as presented in Fig. 2A are boxed in red. (B) Nuclear and cytoplasmic protein fractions were extracted from brain tissue of two oil (Ctrl) or tamoxifen (LoF) treated animals. β-actin was used as loading control after subsequent stripping of the membrane. Lanes presented in Fig. 3A are boxed in red. Figure S3. Vessel diameter changes in SMMHC and GLAST transgenic mice. Quantification of mean vessel diameter in subcortex (A, C) and hippocampus (B, D) of SMMHC- and GLAST-CreERT2; HIF-1αfl/fl mice respectively, treated with oil (Ctrl) or tamoxifen (LoF) and exposed to normoxia or hypoxia (Hx, 8% O2) for 96 h. Two-way ANOVA, mean ± SD, n = 3, *p < 0.05 compared to Nx Ctrl. Figure S4. Vessel diameter changes in GFAP-CreERT2;HIF-1αfl/fl mice. Mean vessel diameter in (A) cortex, (B) subcortex and (C) hippocampus regions of GFAP-CreERT2;HIF-1αfl/fl mice treated with oil (Ctrl) or tamoxifen (Lo [file 12987_2021_302_MOESM1_ESM.pdf]
